# Comparison of the efficacy of taxanes with carboplatin and anthracyclines with taxanes in neoadjuvant chemotherapy for stage II–III triple negative breast cancer: a retrospective analysis

**DOI:** 10.1007/s00432-024-05738-x

**Published:** 2024-06-05

**Authors:** Huibo Wang, Nana Zhang, Qi Sun, Ziqi Zhao, Hui Pang, Xiatian Huang, Ruifeng Zhang, Wenli Kang, Ming Shan

**Affiliations:** 1 Harbin Medical University Cancer Hospital, Harbin Medical University, 150 Haping Road, Nangang District, Harbin, 150081 Heilongjiang China; 2 Beidahuang Group General Hospital, 235 Hashuang Road, Nangang District, Harbin, 150081 Heilongjiang China; 3 Department of Breast Surgery, Harbin Medical University Cancer Hospital, Harbin Medical University, 150 Haping Road, Nangang District, Harbin, 150081 Heilongjiang China

**Keywords:** Neoadjuvant chemotherapy, Triple negative breast cancer, Carboplatin, Pathological complete response, Event-free survival, Overall survival

## Abstract

**Purpose:**

The neoadjuvant chemotherapy (NACT) regimen for triple negative breast cancer (TNBC) primarily consists of anthracyclines and taxanes, and the addition of platinum-based drugs can further enhance the efficacy. However, it is also accompanied by more adverse events, and considering the potential severe and irreversible toxicity of anthracyclines, an increasing number of studies are exploring nonanthracycline regimens that combine taxanes and platinum-based drugs.

**Methods:**

The retrospective study included 273 stage II–III TNBC patients who received NACT. The AT group, consisting of 195 (71.4%) patients, received a combination of anthracyclines and taxanes, while the TCb group, consisting of 78 (28.6%) patients, received a combination of taxanes and carboplatin. Logistic regression analysis was performed to evaluate the factors influencing pathological complete response (pCR) and residual cancer burden (RCB). The log-rank test was used to assess the differences in event-free survival (EFS) and overall survival (OS) among the different treatment groups. Cox regression analysis was conducted to evaluate the factors influencing EFS and OS.

**Results:**

After NACT and surgery, the TCb group had a higher rate of pCR at 44.9%, as compared to the AT group at 31.3%. The difference between the two groups was 13.6% (OR = 0.559, 95% CI 0.326–0.959, *P* = 0.035). The TCb group had a 57.7% rate of RCB 0–1, which was higher than the AT group's rate of 42.6%. The difference between the two groups was 15.1% (OR = 0.543, 95% CI 0.319–0.925, *P* = 0.024), With a median follow-up time of 40 months, the TCb group had better EFS (log-rank, *P* = 0.014) and OS (log-rank, *P* = 0.040) as compared to the AT group. Clinical TNM stage and RCB grade were identified as independent factors influencing EFS and OS, while treatment group was identified as an independent factor influencing EFS, with a close-to-significant impact on OS.

**Conclusion:**

In stage II–III triple TNBC patients, the NACT regimen combining taxanes and carboplatin yields higher rates of pCR and significant improvements in EFS and OS as compared to the regimen combining anthracyclines and taxanes.

## Introduction

Triple negative breast cancer (TNBC) accounts for approximately 15−20% of all molecular subtypes of breast cancer (Zhu et al. 2023). Compared with other types, TNBC is characterized by a higher degree of malignancy, strong invasiveness, and poorer prognosis (Garrido-Castro et al. 2019). Owing to the lack of estrogen receptors (ER), progesterone receptors (PR), and human epidermal growth factor receptor 2 (HER-2), TNBC cannot benefit from endocrine therapy and anti-HER-2 targeted therapy. Chemotherapy remains the primary treatment for TNBC patients, but the optimal chemotherapy regimen has not been clearly defined (Yin et al. 2020).

The treatment approach for stage II–III TNBC has evolved from the previous practice of performing surgery first and then administering adjuvant systemic therapy to the current approach of administering neoadjuvant systemic therapy (NAST) first, followed by surgery (Leon-Ferre and Goetz 2023). This treatment strategy offers several advantages, including tumor size reduction, downstaging of the tumor, understanding drug sensitivity, and providing treatment adjustments based on postoperative pathology, including both escalation and de-escalation of therapy. Patients who achieve pathological complete response (pCR) after NAST experience significant survival benefits as compared to patients with residual disease. In addition, as compared to other subtypes of breast cancer, pCR has greater prognostic value in TNBC patients (Cortazar et al. 2014). Therefore, the primary challenge in the treatment of TNBC is how to select and optimize neoadjuvant chemotherapy (NACT) regimens to achieve higher pCR rates, which can translate into survival benefits.

For a long time, the standard NACT regimen for TNBC has been the combination of anthracyclines and taxanes. However, the addition of platinum-based drugs to this regimen has been shown to further improve patients' pCR rates and long-term survival. This approach has become the preferred NACT backbone for patients suitable for this approach (Poggio et al. 2022). However, the use of multidrug combination therapies inevitably leads to increased toxicity, and anthracycline drugs have unpredictable severe late toxicities (such as cardiac toxicity and hematological disorders) (Tan et al. 2015; Wolff et al. 2015). Therefore, there has been significant interest in exploring regimens without anthracyclines, focusing on the combination of taxanes and platinum-based drugs in the treatment of TNBC. Several prospective and retrospective studies have investigated the efficacy of combining carboplatin with taxanes. It has been observed that this regimen can achieve a good pCR rate and there is no significant difference in long-term survival as compared to regimens that include anthracyclines (Sharma et al. 2017, 2021; Zhang et al. 2022).

To evaluate the therapeutic potential of combining taxanes with carboplatin, we conducted a retrospective analysis of TNBC patients who underwent NACT at our center. These patients were divided into two groups: the taxanes plus carboplatin (TCb) group and the anthracyclines plus taxanes (AT) group. We evaluated the pCR rates in patients treated with two different neoadjuvant groups and compared the rates of event-free survival (EFS) and overall survival (OS) in both groups during follow-up, to determine if there were any differences in long-term prognosis.

## Patient and materials

### Patients

A retrospective selection was conducted on patients who underwent breast cancer surgery and had previously received NACT at Harbin Medical University Cancer Hospital between December 2017 and July 2022. Inclusion criteria included: age over 18 years; histological or cytological confirmation of invasive TNBC; clinical TNM stage II–III; Eastern Cooperative Oncology Group (ECOG) performance status of 0 or 1. Exclusion criteria included: uncertain pathological molecular subtyping; bilateral breast cancer; patients who had already developed or were suspected to have distant metastasis.

### Data collection and definitions

The data on clinical and pathological characteristics were obtained through the review of medical records, including age, body mass index (BMI), menopausal status, family history, Ki-67 index, P53 expression, surgical procedure, chemotherapy regimen, etc. Tumor size, lymph node status, and clinical staging were classified according to the AJCC TNM (8th edition) guidelines (Giuliano et al. 2017). Clinical T stage was determined by combining clinical palpation with comprehensive evaluation through imaging techniques such as ultrasound, mammography, and magnetic resonance imaging (MRI), with the longest diameter of the tumor defining the stage. Clinical N stage was primarily determined based on clinical palpation results. All enrolled patients were followed up via telephone. EFS was defined as the time from the start of NACT to disease recurrence (local recurrence, contralateral recurrence, distant recurrence), primary invasive disease (contralateral breast primary or other second primary cancers), or death from any cause. OS was defined as the time from the start of NACT to death. In cases where disease recurrence or specific death dates could not be determined, the last follow-up date was used as the endpoint for events.

### Pathologic evaluation

All eligible patients underwent a core biopsy of the tumor prior to NACT and underwent a comprehensive pathological evaluation, including assessment of hormone receptors (HR), HER-2 status, Ki-67 index, and P53. ER and PR status were determined using standard immunohistochemistry (IHC) techniques, and nuclear staining of less than 1% was considered negative. HER-2 status was confirmed using either IHC or fluorescence in situ hybridization (FISH). HER-2 IHC scores of 0 and 1 + or nonamplified HER-2 gene by FISH were considered HER-2 negative. The optimal cutoff value for the Ki-67 index in TNBC is yet to be determined. Based on previous research experience, it has been set at 30% (Zhu et al. 2020). According to the standardized definition recommended by NeoSTEEP, pCR in post-NACT surgical patients is defined as the absence of invasive cancer cells in both breast and axillary tissues, or the presence of only residual in situ carcinoma (ypT0/is/ypN0) (Litton et al. 2023). For patients who did not achieve pCR after surgery, IHC testing was performed on breast and axillary specimens to reassess the residual tumor. The residual cancer burden (RCB) assessment system was used to quantify the remaining tumor in the breast and lymph nodes after NACT (Symmans et al. 2007). All pathological results were reviewed and confirmed by two expert pathologists.

### Treatment regimens

We included NACT regimens recommended for TNBC according to breast cancer treatment guidelines in our study. The NACT regimens received by the AT group patients included the combination of taxanes with anthracyclines and cyclophosphamide (TAC), the sequential combination of anthracyclines with cyclophosphamide followed by taxanes (AC-T), and the combination of anthracyclines with taxanes (TA). The TAC regimen consists of intravenous administration of docetaxel (75 mg/m^2^), doxorubicin (50 mg/m^2^), and cyclophosphamide (500 mg/m^2^) every three weeks for a total of six cycles. The AC-T regimen consists of intravenous administration of doxorubicin (90–100 mg/m^2^) and cyclophosphamide (600 mg/m^2^) every three weeks for four cycles, followed by sequential administration of docetaxel (80–100 mg/m^2^) or albumin-bound paclitaxel (125 mg/m^2^) every three weeks for an additional four cycles. The TA regimen involves intravenous administration of doxorubicin (50 mg/m^2^) and docetaxel (75 mg/m^2^) or albumin-bound paclitaxel (125 mg/m^2^) every three weeks. The TCb regimen includes intravenous administration of docetaxel (75 mg/m^2^) or albumin-bound paclitaxel (125 mg/m^2^) and carboplatin (AUC 6 mg/ml/min) every three weeks for a total of six cycles. The drug doses, duration of administration, and number of cycles follow guidelines recommendations. Patients who meet the indications for postoperative radiotherapy receive this treatment, while patients who do not achieve a pCR receive oral capecitabine treatment.

### Statistical analysis

Descriptive statistics were used to analyze patient information and tumor characteristics. The chi-square test or Fisher's exact test was used to compare the balance of baseline characteristics among treatment groups. Logistic regression analysis was used to perform univariate analysis of factors influencing pCR, estimating the odds ratio (OR) and 95% confidence interval for each variable. Variables with a significance level (*P* < 0.10) in the univariate analysis were included as predictive indicators and further analyzed using stepwise logistic regression analysis with the LR method for multivariate logistic regression analysis. In order to minimize the potential bias risk arising from the different treatment durations of the NACT regimens, we decided to evaluate long-term survival starting from the date of surgery. Kaplan–Meier survival curves were used to assess the survival rates and median survival time of different treatment groups. The log-rank test was used to compare the differences in survival time distribution between groups. Univariate and multivariate Cox proportional hazards models were used to evaluate the impact of various factors on EFS and OS. Hazard ratios (HR) and 95% confidence intervals (CI) were calculated for each factor, and independent risk factors with statistically significant differences were selected based on the *P* values. A two-sided *P* value < 0.05 was considered statistically significant. All statistical analyses were performed using IBM SPSS Statistics 26 software (https://www.ibm.com/cn-zh/spss) and R 4.3.0 (https://www.r-project.org/).

## Results

### Patient characteristics

The main baseline characteristics of the patients are shown in Table 1. Our retrospective study ultimately included 273 patients, of whom 195 (71.4%) in the AT group received sequential or combination therapy with anthracyclines and taxanes. 78 (28.6%) patients in the TCb group received combination therapy with taxanes and carboplatin. The median age was 50 years (range 23–72 years). Among the patients, 136 (49.8%) were premenopausal. There were 23 (8.4%) patients with a family history of breast or ovarian cancer. In terms of tumor size, clinical T stage showed 34 (12.5%) patients with cT1 stage, 195 (71.4%) patients with cT2 stage, and 44 (16.1%) patients with cT3–4 stage. Clinical palpation and imaging examinations identified suspicious lymph nodes in 231 (84.6%) patients. 165 (60.4%) patients were confirmed to have axillary or supraclavicular lymph node metastasis through fine-needle aspiration biopsy. 60 (22.0%) patients did not show any evidence of metastasis after the biopsy, and lymph node aspiration was not performed in 48 (17.6%) patients. Close to half of the patients were clinically staged as clinical TNM stage III. All patients were eligible for surgical treatment after NACT. Among them, 218 (79.9%) patients underwent mastectomy, and 42 (15.4%) patients opted for axillary sentinel lymph node biopsy (SLNB) after NACT. There were 96 (35.2%) patients who achieved pCR after surgery, and 128 (46.9%) patients were classified as RCB 0–1. Subsequently, 95 (34.8%) patients received radiation therapy. The proportion of patients with a family history was slightly higher in the TCb group, while other baseline characteristics were well balanced between the two groups.

**Table 1:** Baseline characteristics of patients in the TCb and AT groups

Characteristic	Total (273)	TCb group (78)	AT group (195)	*P* value
Age (years)				0.399
≤ 50	130 (47.6%)	34 (43.6%)	96 (49.2%)	
> 50	143 (52.4%)	44 (56.4%)	99 (50.8%)	
BMI (kg/m^2^)				0.536
< 25	155 (56.8%)	42 (53.8%)	113 (57.9%)	
≥ 25	118 (43.2%)	36 (46.2%)	82 (42.1%)	
Menopausal status				0.301
Premenopausal	136 (49.8%)	35 (44.9%)	101 (51.8%)	
Postmenopausal	137 (50.2%)	43 (55.1%)	94 (48.2%)	
Family history				0.033
No	250 (91.6%)	67 (85.9%)	183 (93.8%)	
Yes	23(8.4%)	11 (14.1%)	12 (6.2%)	
Ki-67				0.518
≤ 30%	74 (27.1%)	19 (24.4%)	55 (28.2%)	
> 30%	199 (72.9%)	59 (75.6%)	140 (71.8%)	
P53				0.731
Negative	126 (46.2%)	30 (44.1%)	78 (41.7%)	
Positive	147 (53.8%)	38 (55.9%)	109 (58.3%)	
Clinical T stage				0.784
T1	34 (12.5%)	8 (10.3%)	26 (13.3%)	
T2	195 (71.4%)	57 (73.1%)	138 (70.8%)	
T3–4	44 (16.1%)	13 (16.7%)	31 (15.9%)	
Clinical N stage				0.410
N0	42 (15.4%)	10 (12.8%)	32 (16.4%)	
N1	106 (38.8%)	35 (44.9%)	71 (36.4%)	
N2–3	125 (45.8%)	33 (42.3%)	92 (47.2%)	
Clinical TNM stage				0.370
IIa	50 (18.3%)	10 (12.8%)	40 (20.5%)	
IIb	88 (32.2%)	30 (38.5%)	58 (29.7%)	
IIIa	111 (40.7%)	31 (39.7%)	80 (41.0%)	
IIIb-c	24 (8.8%)	7 (9.0%)	17 (8.7%)	
Chemotherapy regimen				0.000
TAC	63 (23.1%)	0(0%)	63 (32.3%)	
AC-T	94 (34.4%)	0(0%)	94 (48.2%)	
TA	38 (13.9%)	0(0%)	38 (19.5%)	
TCb	78 (28.6%)	78(100%)	0 (0%)	
Surgery procedure				0.009
Mastectomy	218 (79.9%)	55 (70.5%)	163 (83.6%)	
Breast-conserving + ALND	11 (4.0%)	3 (3.8%)	8 (4.1%)	
Simplemastectomy + SLNB	31 (11.4%)	11 (14.1%)	20 (10.3%)	
Breast-conserving + SLNB	11 (4.0%)	7 (9.0%)	4 (2.1%)	
Reconstruction	2 (0.7%)	2 (2.6%)	0 (0.0%)	
Lymph node status				0.506
Negative	60 (22.0%)	14 (18.0%)	46 (23.6%)	
Positive	165 (60.4%)	48 (61.5%)	117 (60.0%)	
Unknown	48 (17.6%)	16 (20.5%)	32 (16.4%)	
pCR				0.034
pCR	96 (35.2%)	35 (44.9%)	61 (31.3%)	
Non-pCR	177 (64.8%)	43 (31.3%)	134 (68.7%)	
RCB				0.024
RCB 0–1	128 (46.9%)	45 (57.7%)	83 (42.6%)	
RCB 2–3	145 (53.1%)	33 (42.3%)	112 (57.4%)	
Radiotherapy				0.278
No	178 (65.2%)	47 (60.3%)	131 (67.2%)	
Yes	95 (34.8%)	31 (39.7%)	64 (32.8%)	

*T* taxanes, *A* anthracyclines, *C* cyclophosphamide, *TCb* carboplatin, *BMI* body mass index, *ALND* axillary lymph node dissection, *SLNB* sentinel lymph node biopsy, *pCR* pathological complete response, *RCB* residual cancer burden, *Lymph Node Status* The Lymph Node Status refers to the situation at the time of biopsy before neoadjuvant therapy

### Pathological response

**Fig. 1:**
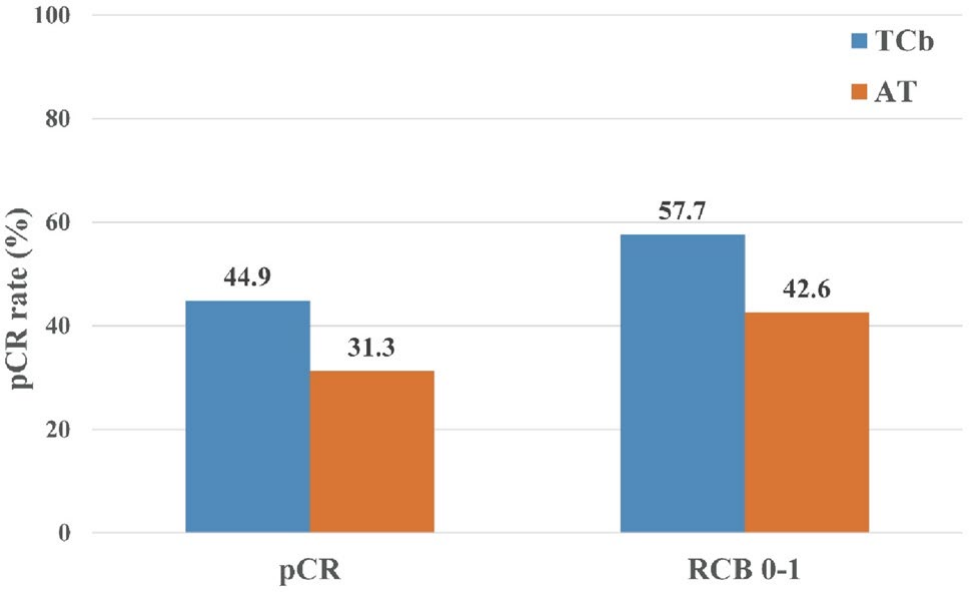
The treatment group achieved a rate of achieving pCR and RCB 0–1

After NACT and postoperative pathological analysis (Fig. 1), 35 (44.9%) patients in the TCb group achieved pCR, while 61 (31.3%) patients in the AT group achieved pCR, with a difference of 13.6% (OR = 0.559, 95% CI 0.326–0.959, *P* = 0.035). In terms of RCB 0–1, the TCb group had 45 (57.7%) patients, while the AT group had 83 (42.6%) patients, with a difference of 15.1% (OR = 0.543, 95% CI 0.319–0.925, *P* = 0.024).

As shown in Table 2, the univariate logistic regression analysis of factors influencing the pCR rate demonstrated that lower clinical T stage and the TCb group increased the likelihood of achieving pCR in patients (*P* = 0.014 and *P* = 0.035, respectively). These associations remained statistically significant in the multivariate logistic regression analysis (*P* = 0.011 and *P* = 0.024, respectively). Furthermore, the univariate and multivariate logistic regression analyses of factors influencing RCB 0–1, as shown in Table 3, revealed that clinical T stage and the treatment group were also independent predictors of RCB 0–1, similar to their effects on pCR (*P* = 0.004 and *P* = 0.020, respectively). In addition, a Ki-67 index > 30% was significantly associated with RCB 0–1 (*P* = 0.012).

**Table 2:** Univariate and multivariate logistic regression analysis for pCR

	pCR (96)	Non-pCR (177)	OR	95% CI	*P* value	*P* value^a^
AGE (years)			0.711	0.432–1.171	0.180	
≤ 50	51 (53.1%)	79 (44.6%)				
> 50	45 (46.9%)	98 (55.4%)				
BMI (kg/m^2^)			1.034	0.626–1.706	0.897	
≤ 25	54 (56.2%)	101 (57.1%)				
> 25	42 (43.8%)	76 (42.9%)				
Menopausal status			0.716	0.435–1.180	0.190	
Premenopausal	53 (55.2%)	83 (46.9%)				
Postmenopausal	43 (44.8%)	94 (53.1%)				
Family history			0.627	0.239–1.648	0.344	
No	90 (93.8%)	160 (90.4%)				
Yes	6 (6.2%)	17 (9.6%)				
Ki-67			1.668	0.927–3.002	0.088	
≤ 30%	20 (20.8%)	54 (30.5%)				
> 30%	76 (79.2%)	123 (69.5%)				
P53			0.692	0.420–1.140	0.149	
Negative	43 (48.3%)	65 (39.2%)				
Positive	46 (51.7%)	101 (60.8%)				
Clinical T stage						
T1	18 (18.8%)	16 (9.0%)			0.014	0.011
T2	69 (71.9%)	126 (71.2%)	0.487	0.233–1.015	0.055	0.042
T3–4	9 (9.4%)	35 (19.8%)	0.229	0.085–0.618	0.004	0.003
Clinical N stage						
N0	16 (16.7%)	26 (14.7%)			0.165	
N1	30 (31.2%)	76 (42.9%)	0.641	0.302–1.362	0.248	
N2–3	50 (52.1%)	75 (42.4%)	1.083	0.528–2.222	0.827	
Clinical TNM stage						
IIa	19 (19.8%)	31 (17.5%)			0.403	
IIb	25 (26.0%)	63 (35.6%)	0.647	0.310–1.351	0.247	
IIIa	44 (45.8%)	67 (37.9%)	1.071	0.540–2.128	0.844	
IIIb–c	8 (8.3%)	16 (9.0%)	0.816	0.293–2.269	0.696	
Treatment group			0.559	0.326–0.959	0.035	0.024
TCb	35 (36.5%)	43 (24.3%)				
AT	61 (63.5%)	134 (75.7%)				

*pCR* pathological complete response, *BMI* body mass index, *TCb* TCb group, *AT* AT group, *OR* odds ratio

^a^Multivariate analysis is significant

**Table 3:** Univariate and multivariate logistic regression analysis for RCB 0–1

	RCB 0–1 (128)	RCB2-3 (145)	OR	95% CI	*P* value	*P* value^a^
Age (years)			0.699	0.434–1.128	0.142	
≤ 50	67 (52.3%)	63 (43.4%)				
> 50	61 (47.7%)	82 (56.6%)				
BMI (kg/m^2^)			0.726	0.448–1.176	0.193	
≤ 25	78 (60.9%)	77 (53.1%)				
> 25	50 (39.1%)	68 (46.9%)				
Menopausal status			0.734	0.456–1.183	0.205	
Premenopausal	69 (53.9%)	67 (46.2%)				
Postmenopausal	59 (46.1%)	78 (53.8%)				
Family history			0.860	0.364–2.035	0.732	
No	118 (92.2%)	132 (91.0%)				
Yes	10 (7.8%)	13 (9.0%)				
Ki-67			2.103	1.206–3.668	0.009	0.012
≤ 30%	25 (19.5%)	49 (33.8%)				
> 30%	103 (80.5%)	96 (66.2%)				
P53			0.841	0.522–1.355	0.477	
Negative	62 (48.4%)	64 (44.1%)				
Positive	66 (51.6%)	81 (55.9%)				
Clinical T stage						
T1	23 (18.0%)	11 (7.6%)			0.005	0.004
T2	92 (71.9%)	103 (71.0%)	0.427	0.197–0.924	0.031	0.038
T3–4	13 (10.2%)	31 (21.4%)	0.201	0.076–0.528	0.001	0.003
Clinical N stage						
N0	22 (17.2%)	20 (13.8%)			0.581	
N1	46 (35.9%)	60 (41.4%)	0.697	0.340–1.428	0.324	
N2–3	60 (46.9%)	65 (44.8%)	0.839	0.417–1.690	0.839	
Clinical TNM stage						
IIa	27 (21.1%)	23 (15.9%)			0.700	
IIb	41 (32.0%)	47 (32.4%)	0.743	0.370–1.491	0.403	
IIIa	50 (39.1%)	61 (42.1%)	0.698	0.357–1.365	0.293	
IIIb–c	10 (7.8%)	14 (9.7%)	0.608	0.228–1.627	0.322	
Treatment group			0.543	0.319–0.925	0.024	0.020
TCb	45 (35.2%)	33 (22.8%)				
AT	83 (64.8%)	112 (77.2%)				

*pCR* pathological complete response, *BMI* body mass index, *TCb* TCb group, *AT* AT group, *OR* odds ratio

^a^Multivariate analysis is significant

### Survival analysis

From the start of treatment to the end of the follow-up period, the median follow-up time was 40 months, with an average follow-up time of 36.5 months (range 5–61 months). We observed 44 (16.1%) cases of EFS events and 30 (11.0%) cases of OS events.

**Fig. 2:**
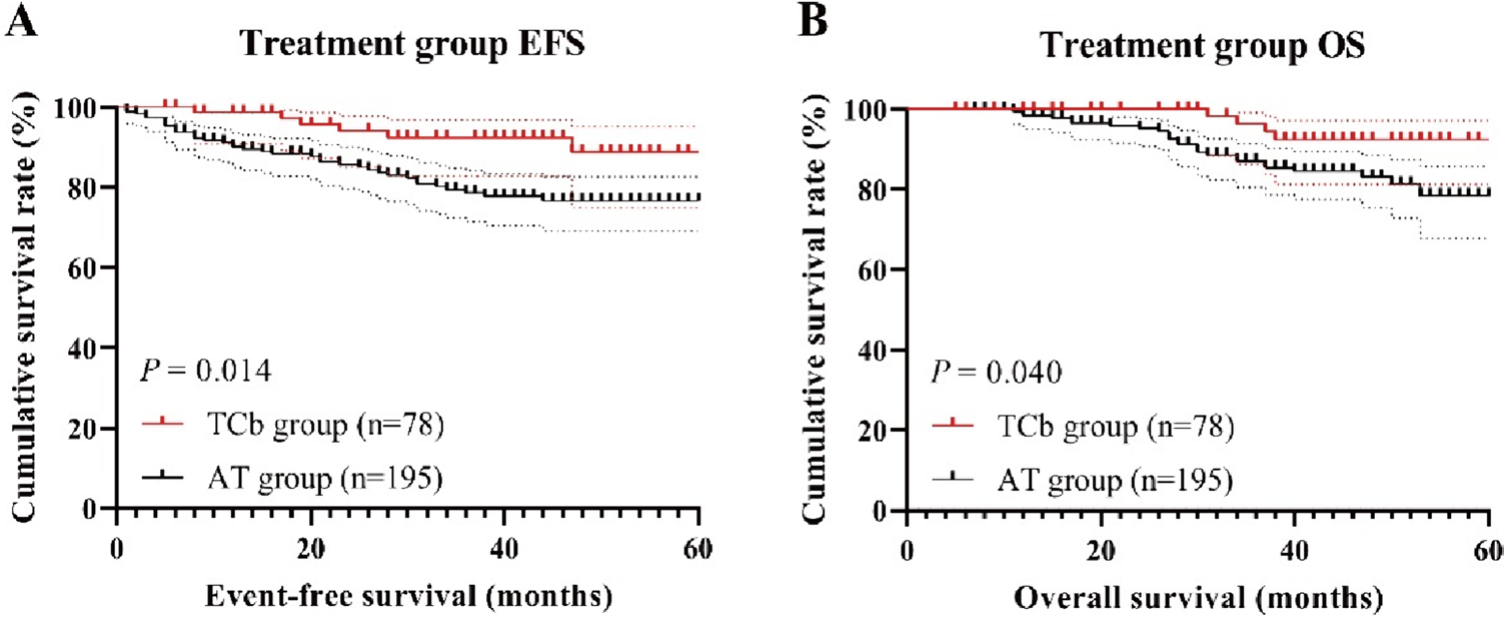
The Kaplan–Meier survival curves for EFS (**A**) and OS (**B**) in the treatment group

The Kaplan–Meier curves for EFS and OS in the treatment group (Fig. 2A, B). The TCb group and AT group did not reach the median survival time for both EFS and OS. Patients in the TCb group had a better survival trend compared to patients in the AT group, with a 3-year EFS rate of 92.5% and 78.6% respectively (log-rank, *P* = 0.014), and a 3-year OS rate of 96.4% and 87.0% respectively (log-rank, *P* = 0.040).

**Fig. 3:**
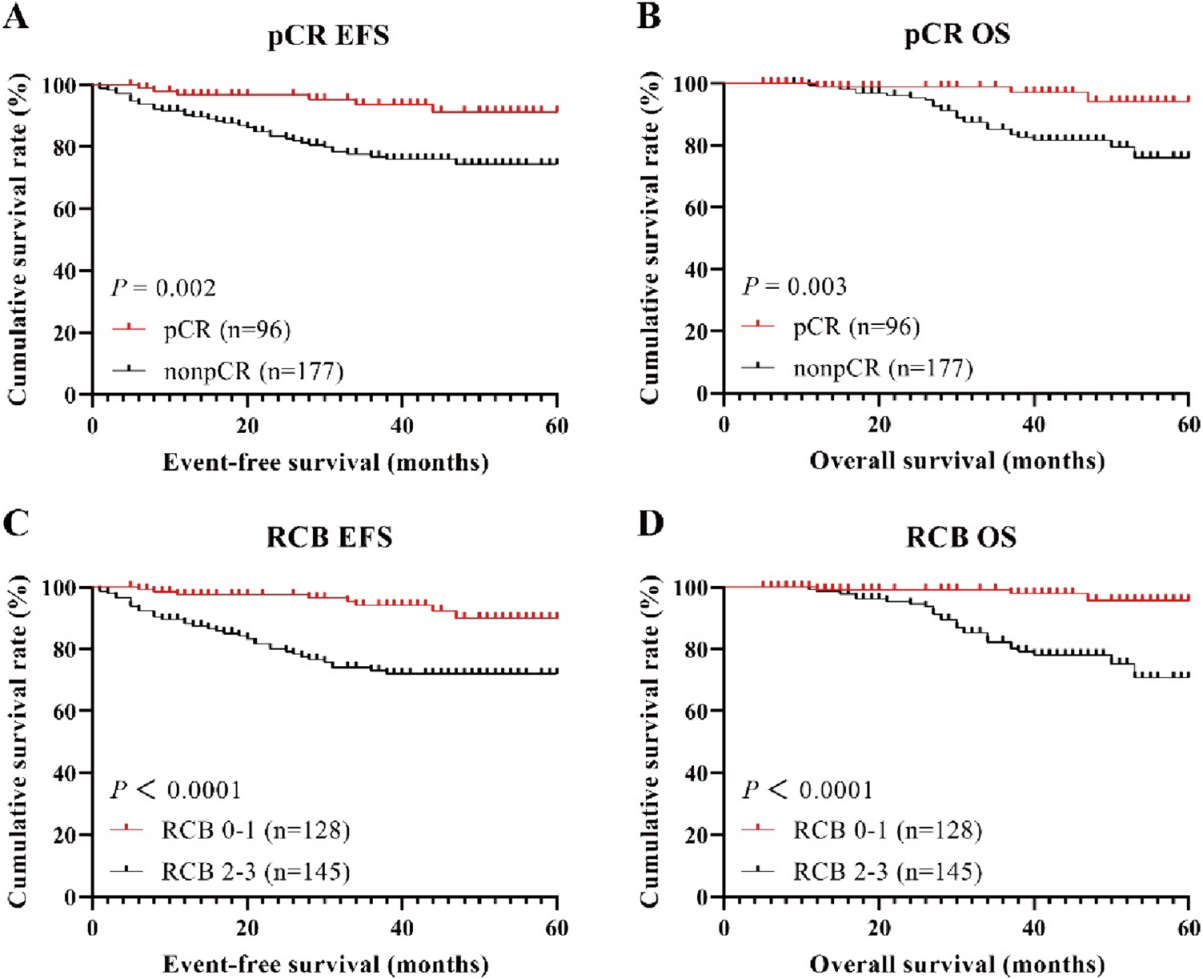
The Kaplan–Meier survival curves for EFS (**A**, **C**) and OS (**B**, **D**) in the pCR and RCB groups

Patients who achieved a pCR had significantly prolonged survival time as compared to those who did not achieve pCR, both in terms of EFS and OS (Fig. 3A, B). The 3-year EFS rate for pCR patients was 93.6% as compared to 76.8% for non-pCR patients (log-rank, *P* = 0.002), and the 3-year OS rate was 98.9% for pCR patients as compared to 85.1% for non-pCR patients (log-rank, *P* = 0.003). The comparison of EFS and OS between RCB 0–1 and RCB 2–3 patients also showed significant survival benefits (Fig. 3C, D). The 3-year EFS rates were 94.1% for RCB 0–1 patients and 73.0% for RCB 2–3 patients (log-rank, *P* < 0.0001), while the 3-year OS rates were 99.2% for RCB 0–1 patients and 82.3% for RCB 2–3 patients (log-rank, *P* < 0.0001).

**Fig. 4:**
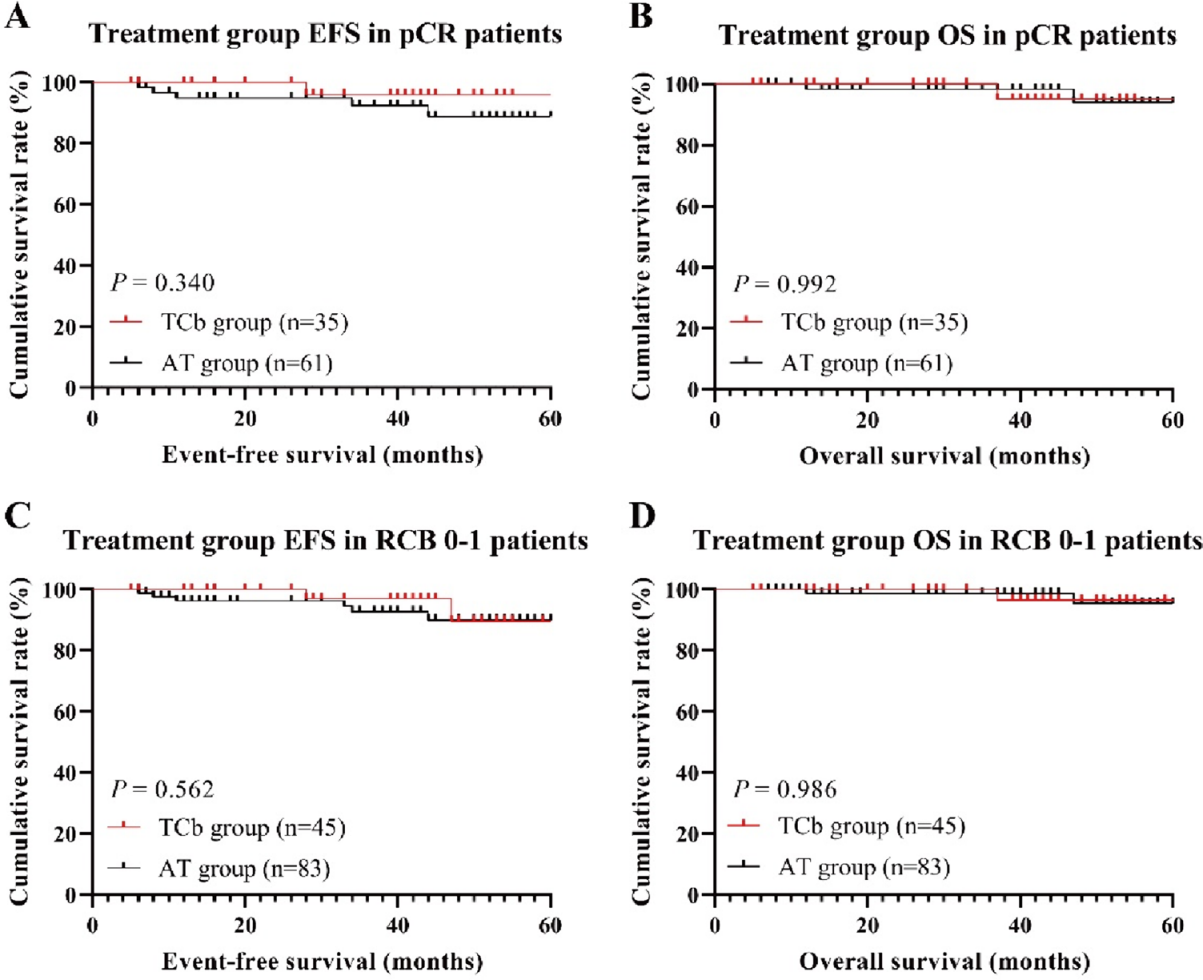
The Kaplan–Meier survival curves for EFS (**A**, **C**) and OS (**B**, **D**) in the pCR and RCB 0–1 subgroup of the treatment group

**Fig. 5:**
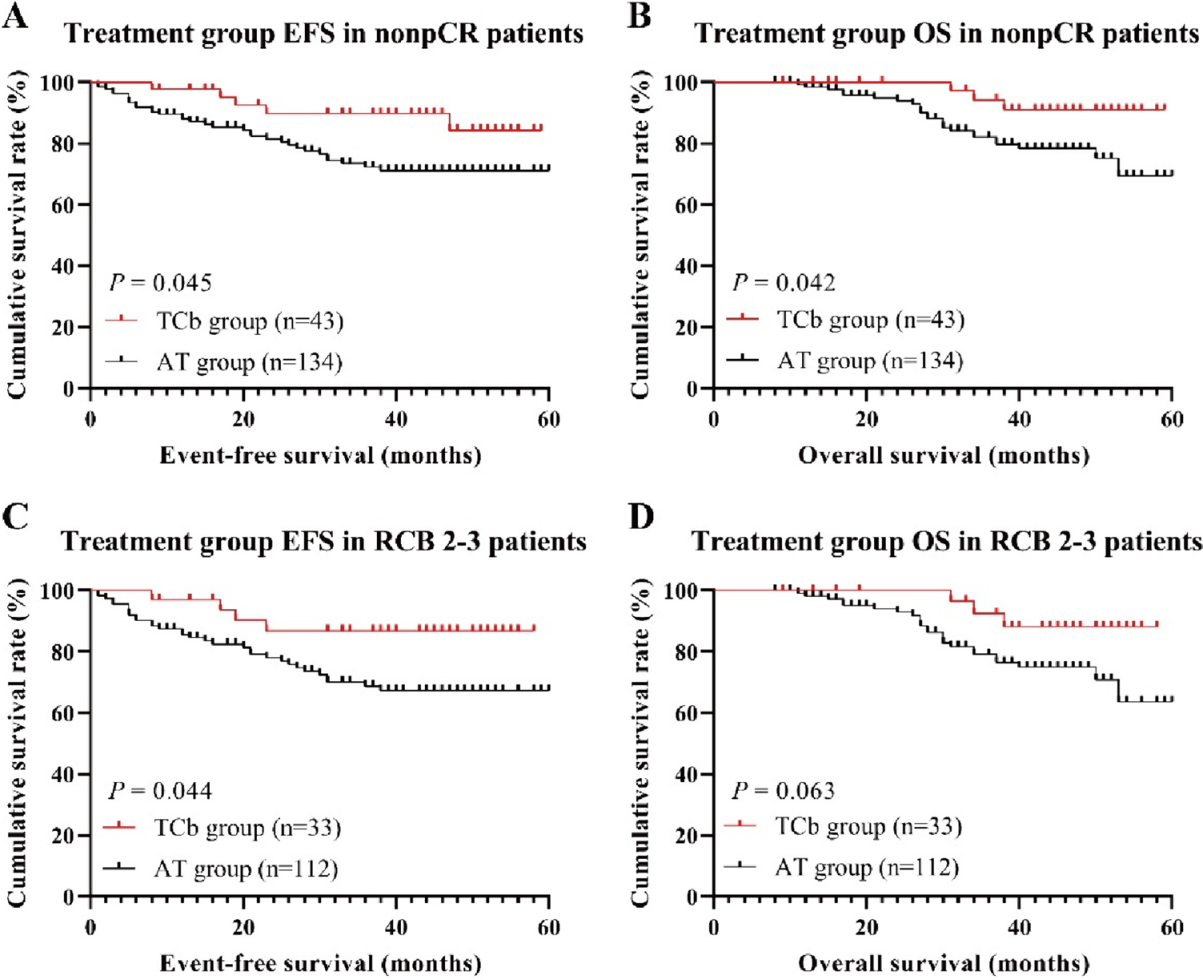
The Kaplan–Meier survival curves for EFS (**A**, **C**) and OS (**B**, **D**) in the nonpCR and RCB 2–3 subgroup of the treatment group

Among patients who achieved pCR and RCB 0–1, there were no significant differences in EFS and OS between different treatment groups (pCR: log-rank, *P* = 0.340 and *P* = 0.992, respectively; RCB 0–1: log-rank, *P* = 0.562 and *P* = 0.986, respectively). The 3-year EFS and OS rates exceeded 90% in all treatment groups (Fig. 4). In non-pCR patients, there were significant statistical differences in EFS and OS between treatment groups (Fig. 5A, B). The 3-year EFS rates for the TCb group and AT group were 89.8% and 72.3% respectively (log-rank, *P* = 0.045), while the 3-year OS rates were 94.2% and 82.0% respectively (log-rank, *P* = 0.042). In RCB 2–3 patients, there were significant statistical differences in EFS between treatment groups, but the differences in OS were not significant (Fig. 5C, D). The 3-year EFS rates for the TCb group and AT group were 86.8% and 68.7% respectively (log-rank, *P* = 0.044), while the 3-year OS rates were 92.4% and 79.1% respectively (log-rank, *P* = 0.063).

The univariate cox regression analysis for EFS, as shown in Table 4, indicates that clinical N stage, clinical TNM stage, treatment group, pCR, and RCB grade are significant factors associated with EFS. When including these factors in a multivariable cox regression analysis, except for clinical TNM stage and RCB grade, the treatment group remains an independent factor influencing EFS. The TCb group shows a significant improvement in EFS compared to the AT group (HR = 2.587; 95% CI 1.083–6.175; *P* = 0.032). The univariate cox regression analysis for OS, as shown in Table 5, indicates that clinical T stage, clinical N stage, clinical TNM stage, pCR, and RCB grade are significant factors associated with OS. The treatment group is borderline significant (*P* = 0.051). In the multivariable cox regression analysis, similar to the independent factors for EFS, clinical TNM stage and RCB grade are independent factors for OS. The treatment regimen approaches statistical significance (HR = 2.854; 95% CI 0.980–8.311; *P* = 0.054).

**Table 4:** Univariate and multivariate cox regression analysis for EFS

	Univariate		Multivariate	
	HR (95% CI)	*P*	HR (95% CI)	*P*
Age (years)				
≤ 50	Ref			
> 50	1.320 (0.724–2.409)	0.365		
BMI (kg/m^2^)				
≤ 25	Ref			
> 25	0.853 (0.467–1.555)	0.603		
Menopausal status				
Premenopausal	Ref			
Postmenopausal	1.057 (0.585–1.910)	0.854		
Family history				
No	Ref			
Yes	1.418 (0.559–3.598)	0.462		
Ki-67				
≤ 30%	Ref			
> 30%	0.700 (0.379–1.295)	0.256		
P53				
Negative	Ref			
Positive	0.811 (0.449–1.465)	0.487		
Clinical T stage				
T1	Ref	0.308		
T2	1.277 (0.450–3.626)	0.646		
T3–4	2.102 (0.659–6.703)	0.209		
Clinical N stage				
N0	Ref	0.042		
N1	2.952 (0.678–12.844)	0.149		
N2–3	4.934 (1.171–20.791)	0.030		
Clinical TNM stage				
IIa	Ref	0.004	Ref	0.004
IIb	1.152 (0.400–3.317)	0.793	1.213 (0.420–3.503)	0.722
IIIa	1.830 (0.683–4.903)	0.229	1.765 (0.658–4.733)	0.259
IIIb–c	5.097 (1.704–15.248)	0.004	5.271 (1.752–15.861)	0.003
Treatment group				
TCb	Ref		Ref	
AT	2.812 (1.188–6.653)	0.019	2.587 (1.083–6.175)	0.032
pCR				
Non-pCR	Ref			
pCR	0.274 (0.116–0.649)	0.003		
RCB				
RCB 0–1	Ref		Ref	
RCB 2–3	4.283 (1.990–9.214)	0.000	3.779 (1.750–8.165)	0.001
Radiotherapy				
No	Ref			
Yes	1.429 (0.789–2.589)	0.238		

*EFS* event-free survival, *HR* hazard ratio, *BMI* body mass index, *pCR* pathological complete response, *RCB* residual cancer burden

**Table 5:** Univariate and multivariate cox regression analysis for OS

	Univariate		Multivariate	
	HR (95% CI)	*P*	HR (95% CI)	*P*
Age (years)				
≤ 50	Ref			
> 50	1.793 (0.839–3.832)	0.132		
BMI (kg/m^2^)				
≤ 25	Ref			
> 25	0.706 (0.336–1.484)	0.759		
Menopausal status				
Premenopausal	Ref			
Postmenopausal	1.220 (0.592–2.512)	0.590		
Family history				
No	Ref			
Yes	1.645 (0.574–4.715)	0.354		
Ki-67				
≤ 30%	Ref			
> 30%	0.503 (0.245–1.030)	0.060		
P53				
Negative	Ref			
Positive	1.233 (0.599–2.540)	0.570		
Clinical T stage				
T1	Ref	0.044		
T2	1.713 (0.399–7.356)	0.469		
T3–4	4.229 (0.912–19.612)	0.065		
Clinical N stage				
N0	Ref	0.015		
N1	2.921 (0.370–23.082)	0.309		
N2–3	7.528 (1.009–56.171)	0.049		
Clinical TNM stage				
IIa	Ref	0.000	Ref	0.000
IIb	0.984 (0.246–3.945)	0.984	1.116 (0.277–4.496)	0.878
IIIa	2.168 (0.617–7.623)	0.228	2.037 (0.651–8.181)	0.195
IIIb-c	8.781 (2.298–33.553)	0.001	10.018 (2.565–39.119)	0.001
Treatment group				
TCb	Ref		Ref	
AT	2.858 (0.997–8.190)	0.051	2.854 (0.980–8.311)	0.054
pCR				
Non-pCR	Ref			
pCR	0.197 (0.060–0.651)	0.008		
RCB				
RCB 0–1	Ref		Ref	
RCB 2–3	8.294 (2.513–27.377)	0.001	7.421 (2.238–24.605)	0.001
Radiotherapy				
No	Ref			
Yes	1.568 (0.766–3.208)	0.218		

*OS* overall survival, *HR* hazard ratio, *BMI* body mass index, *pCR* pathological complete response, *RCB* residual cancer burden

## Discussion

Breast cancer, as the cancer with the highest global incidence, seriously threatens the lives and health security of women. TNBC as the most aggressive and poorest prognosis molecular subtype, has always had unmet treatment needs (Sung et al. 2020). The current main issue is how to improve the efficacy of NACT, which has already been established as the standard treatment approach for stage II–III TNBC (Harbeck et al. 2019). In recent years, the efficacy and prognosis of NAST for TNBC have been continuously improved through the selection and combination of chemotherapy drugs, as well as the development and application of immune check-point inhibitors (ICIs), PARP inhibitors, antibody–drug conjugates (ADCs), and other promising drugs. However, cytotoxic chemotherapy remains the cornerstone of standard treatment (Bianchini et al. 2022).

NACT regimens based on anthracyclines and taxanes remain the preferred options according to guidelines (Gradishar et al. 2023). However, there are also studies in stage II–III TNBC that have incorporated platinum-based drugs into neoadjuvant regimens with taxanes and an-thracyclines, further improving patients' pCR rates and survival benefits. In the GeparSixto randomized phase II trial, among TNBC patients, those who received carboplatin treatment showed a significant increase in pCR rates as compared to those who did not receive carboplatin treatment (Minckwitz et al. 2014). Moreover, the survival data with a median follow-up time of 47.3 months observed better disease-free survival (DFS) benefits in the platinum-containing group. However, the improvement in OS did not reach statistical significance (Loibl et al. 2018a). In the CALGB 40603 trial, the addition of carboplatin also resulted in a higher pCR rate. However, in the survival analysis with a median follow-up time of 7.9 years, the addition of carboplatin did not show a significant improvement in EFS, and there was also no significant improvement in OS (Minckwitz et al. 2014; Shepherd et al. 2022). Subsequently, a phase III randomized clinical trial, BrighTNess, demonstrated that the addition of carboplatin to sequential anthracycline followed by taxane can improve patients' pCR rates. However, the further addition of veliparib did not lead to a significant increase in pCR rates (Loibl et al. 2018b). Excitingly, at a median follow-up time of 4.5 years, the addition of carboplatin improved the EFS of TNBC patients. Furthermore, the additional inclusion of veliparib did not result in improved EFS. This may suggest that the improvement in pCR and EFS in TNBC patients is primarily attributed to the addition of carboplatin (Geyer et al. 2022). The latest meta-analysis also indicates that adding platinum to regimens based on anthracyclines and taxanes can improve patients' pCR rates and increase their EFS, making it a possible option for NACT in TNBC (Poggio et al. 2022; Li et al. 2020).

The KEYNOTE-522 study evaluated the combination of immunotherapy and cytotoxic chemotherapy in neoadjuvant treatment for TNBC patients, which significantly improved patients' pCR rates and EFS, thus changing the treatment paradigm for early-stage TNBC patients (Schmid et al. 2020, 2022). The study selected a highly responsive chemotherapy regimen, including anthracyclines, taxanes, platinum, and cyclophosphamide, to determine the advantages of immunotherapy while also addressing the long-standing issue of the use of platinum-based drugs in NACT for TNBC patients. However, it is undeniable that the use of more cytotoxic drugs is associated with increased toxicity (Leon-Ferre and Goetz 2023). Moreover, in the context of immunotherapy, the true efficacy and survival benefits of the four-drug combination chemotherapy regimen in neoadjuvant treatment for TNBC patients are uncertain. Due to the irreversible and severe cardiotoxicity and hematotoxicity that anthracyclines may cause, an increasing number of studies suggest that a two-drug combination regimen consisting of taxanes and platinum may be a better choice for NACT in TNBC patients (Tan et al. 2015; Wolff et al. 2015). A combined analysis of two cohorts indicated that neoadjuvant treatment with carboplatin combined with docetaxel achieved a pCR rate of 55% and an RCB 0 + 1 rate of 68% in TNBC patients. The treatment also demonstrated good tolerability (Sharma et al. 2017). A phase II clinical trial study on NACT for operable breast cancer demonstrated that the use of dose-dense paclitaxel combined with carboplatin achieved a pCR rate of 57.14% in the triple-negative subgroup. However, it should be noted that there were only 14 patients in the triple-negative subgroup (Zhu et al. 2016). In the selection of neoadjuvant treatment regimens for stage I–III TNBC patients, the randomized phase II clinical study NeoSTOP compared a two-drug regimen of docetaxel combined with carboplatin to a four-drug regimen of sequential doxorubicin and cyclophosphamide followed by paclitaxel combined with carboplatin. The results showed no significant difference in pCR rates and RCB 0–1 probabilities between the two groups. At a median follow-up of 38 months, the two groups had similar EFS and OS rates. The two-drug regimen had a lower incidence of 3/4 adverse events and lower treatment costs (Sharma et al. 2021). In another phase II study, NeoCART, a comparison was made between a two-drug regimen of docetaxel plus carboplatin and a three-drug regimen of epirubicin plus cyclophosphamide followed by docetaxel. The results showed that the two-drug regimen achieved a higher pCR rate. At a median follow-up of 37 months, the two groups had similar EFS and OS rates (Zhang et al. 2022). It is worth noting that there was one case of fatal secondary leukemia in the group treated with anthracycline. However, there have been no studies that have found long-term survival improvement in the regimen of taxanes combined with carboplatin as compared to the regimen of anthracyclines combined with taxanes.

To further investigate the aforementioned question, we conducted a retrospective analysis at our center, comparing the taxanes plus carboplatin (TCb) group with the anthracycline plus taxanes (AT) group. Our results showed that the TCb group had higher pCR rates and better long-term survival as compared to the AT group. It is worth noting that in our study, over 80% of the enrolled patients had clinically lymph node-positive disease, and over 60% of the patients were confirmed to have lymph node metastasis through fine-needle aspiration biopsy. Compared with the previous neoadjuvant trial populations, our study had a higher proportion of patients with lymph node-positive disease. This may explain why our study, which used a similar TCb regimen compared to the NeoCART and NeoSTOP studies, had numerically lower pCR rates. Consistent with the previous research findings, patients who achieved pCR after neoadjuvant treatment showed significant improvement in EFS and OS as compared to those who did not achieve pCR (Spring et al. 2020). Our results also confirmed this, and we found that the RCB classification significantly influenced EFS and OS. In fact, our analysis revealed that RCB classification had a better predictive value for survival as compared to pCR. Unlike the previous studies, our research found that at a median follow-up of 40 months, the TCb group showed a significant improvement in EFS and OS as compared to the AT group. In addition, we observed a significant improvement in EFS and OS in the population with non-pCR and with RCB 2–3 after NACT and surgery. These results may suggest that the TCb regimen has a better efficacy and potential for improving long-term survival in neoadjuvant treatment of high-risk and resistant TNBC patients.

Our study has certain limitations. Firstly, it is a single-center retrospective study and not a prospective randomized controlled trial. There was an imbalance in the number of patients between the treatment groups, but the main baseline characteristics between the two groups were balanced. In addition, the patients receiving the TCb regimen were based on the treatment preferences of certain doctors, and there was no patient selection bias. It is worth mentioning that the average and median follow-up times were similar between the two treatment groups, and there was no time difference in the initiation of treatment between the groups. Secondly, our treatment regimen primarily followed a three-week cycle and did not select the shorter cycle preferred by the guidelines, similar to the treatment cycles in the KEYNOTE-522, NeoSTOP, and NeoCART trials. Thirdly, the retrospective data we collected did not record adverse reactions related to the treatment regimens, so toxicity assessment could not be performed. However, our main objective was to explore the efficacy of the TCb regimen, and previous studies have indicated that the TCb regimen is well tolerated.

## Conclusion

In conclusion, our study demonstrates that the combination of taxanes with carboplatin in NACT for TNBC leads to higher pCR rates and significant improvements in EFS and OS as compared to the combination of anthracyclines with taxanes. Further randomized controlled trials are needed to evaluate the efficacy of the combination of taxanes and carboplatin, as well as the potential of this regimen as a new chemotherapy backbone in combination with immunotherapy for treatment.

## Data Availability

The datasets generated during and/or analysed during the current study are available from the corresponding author on reasonable request.
